# Physical Issues in Mental Health Settings – Implementation of a New Immersive Course for Core Psychiatry Trainees

**DOI:** 10.1192/bjo.2024.314

**Published:** 2024-08-01

**Authors:** Vatsala Mishra, Ian Winston

**Affiliations:** Barts Health NHS Trust, London, United Kingdom

## Abstract

**Aims:**

This team of simulation fellows at a London teaching hospital created an immersive simulation course for core psychiatry trainees to explore the intersection between physical and mental health and impact on provision of care. The course was fully mapped to the updated Royal College Core Training Curriculum as well as Crisis Resource Management principles, and focusses on the integration of care across mental and physical health provisions. Scenarios are set in a range of inpatient and community environments to allow participants to consider differences in delivery of holistic care, prioritisation, ethical and legal considerations across settings. This would be particularly relevant for participants early in training with limited prior exposure who may be unfamiliar with handling emergencies on psychiatric wards and the nuanced limitations in providing medical care.

**Methods:**

Scenarios were written in consultation with speciality experts and allied health professionals including mental health nursing, dietetics, and pharmacy. The course is written to enable participants to explore the intersection between physical and mental health, and the practical and social implications of an individual's mental and physical condition on provision of care. Alongside debriefing technical and non-technical learning objectives, participants reflected upon the wider determinants of each patient's current physical and mental state and discussed ethicolegal considerations such as patients’ legal status, capacity to consent, and practicalities of transferring patients between services and facilitating holistic care.

**Results:**

The pilot course took place on July 4^th^ following consultation with stakeholders including senior simulation and education leads within the Trust, and deanery Training Programme Directors, to ensure the course was formally endorsed to allow participants to apply for study leave to attend. Post-course feedback was collected through use of Likert-scales and white space questions; the response was highly positive and showed the programme met its aims and filled a training need. Feedback showed increased confidence managing integrated physical and mental health issues and balancing conflicting priorities with increased understanding of practical and social implications of mental and physical condition on provision of care.

**Conclusion:**

Next steps involve collaboration with service users to allow accurate representation of the unique needs of a diverse population, and potential use of actors to sensitively and ethically portray simulated patients. Local psychiatry training schools could be approached to consider formal implementation of the course within academic programmes, in addition to potential reformulation of scenarios for use in established courses at the host site such as Undergraduate or Foundation training days.

